# Osteosarcoma of the Right Lower Femur With Breast and Axillary Lymph Node Metastasis: A Rare Case Presentation

**DOI:** 10.7759/cureus.20813

**Published:** 2021-12-29

**Authors:** Bijayalaxmi Sahoo, Sandip Barik, Sujata Naik, Saroj Kumar Das Majumdar, Dillip Kumar Parida

**Affiliations:** 1 Radiation Oncology, All India Institute of Medical Sciences, Bhubaneswar, IND; 2 Pathology, Institute of Medical Sciences and SUM Hospital, Siksha 'O' Anusandhan (Deemed to be University), Bhubaneswar, IND

**Keywords:** metastasis, immunohistochemistry, axillary lymph node, osteosarcoma, breast metastasis

## Abstract

Osteosarcoma is the most common skeletal malignancy and commonly metastasis to lung and bone. Here we report a case of osteosarcoma of the right knee with metastasis to the lower and inner quadrant of the breast along with axillary, mediastinal, retroperitoneal and inguinal lymphadenopathy with lung and liver metastasis. The diagnosis of breast metastasis was confirmed by ultrasonography-guided biopsy and immunohistochemistry (IHC). So this report highlights the rarest metastasis to breast and axillary lymph node from an osteosarcoma of the right knee primary.

## Introduction

Osteosarcoma is the most common primary bone malignancy [1]. Osteosarcoma mostly metastasizes to the lung followed by the bone. Breast metastasis from extra mammary malignancies is uncommon as most of the metastasis to the breast is from contralateral breast primaries [2-3]. So osteosarcoma metastasis to the breast is extremely rare [4-5]. Here we report a case of a 20-year-old female patient with metastatic osteosarcoma of the right knee with metastasis to the breast and axillary lymph node; we also focus on the diagnosis and management of this case.

## Case presentation

**Figure 1:**
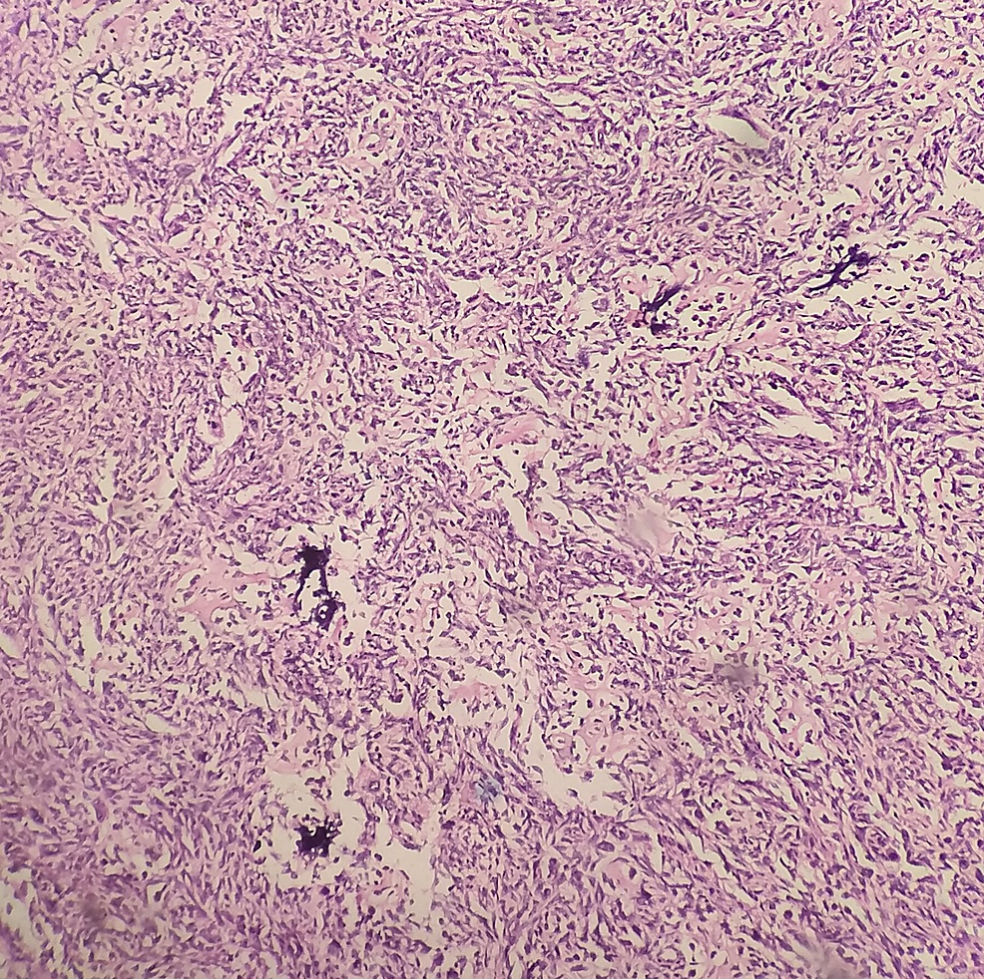
100x hematoxylin and eosin (H&E) microscope image showing neoplastic spindloid cells arranged in a herring bone pattern with the presence of malignant osteoid

**Figure 2:**
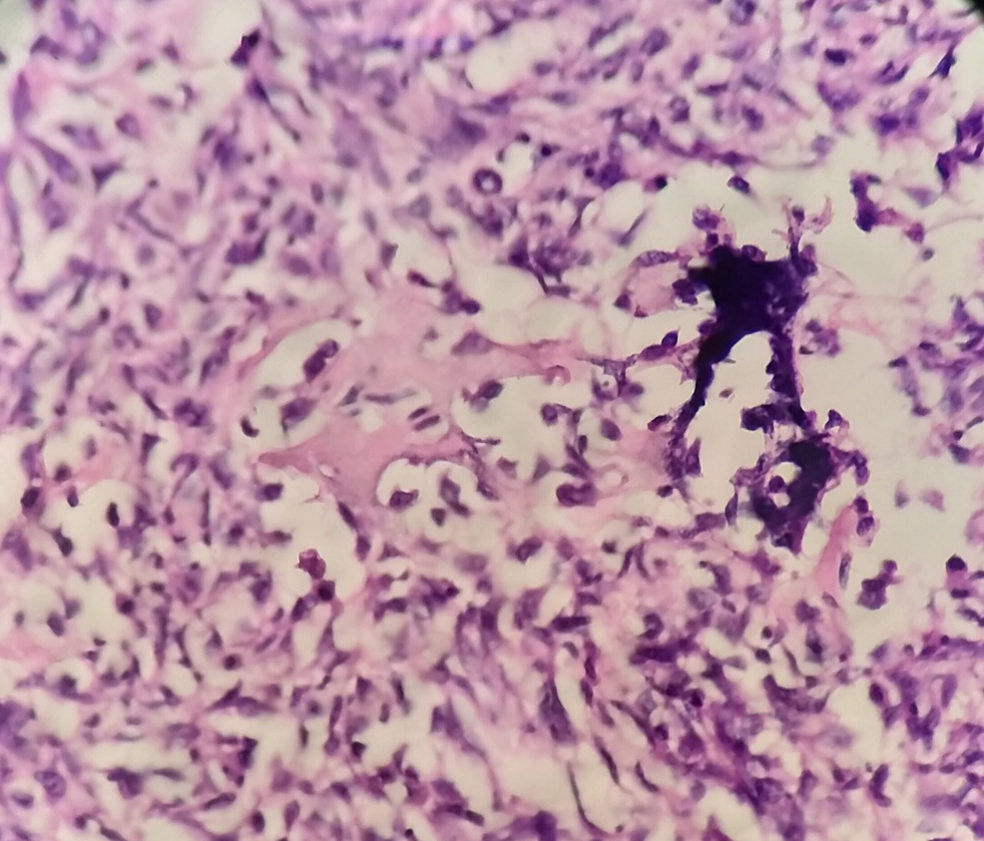
400x hematoxylin and eosin (H&E) microscope feature showing presence of malignant matrix, tumor giant cells, and mitosis

**Figure 3:**
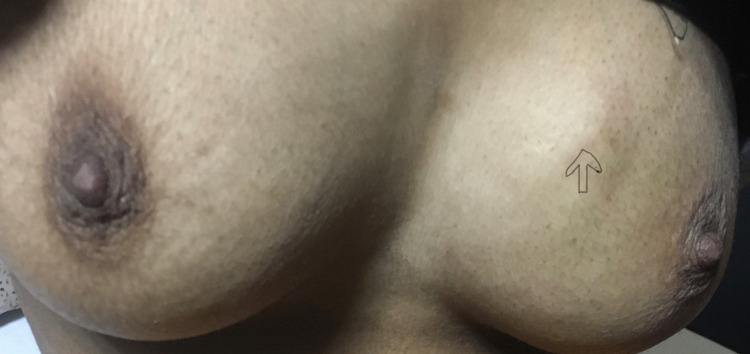
Hard lump of size 3 x 3 cm over the inner quadrant of the left breast

**Figure 4:**
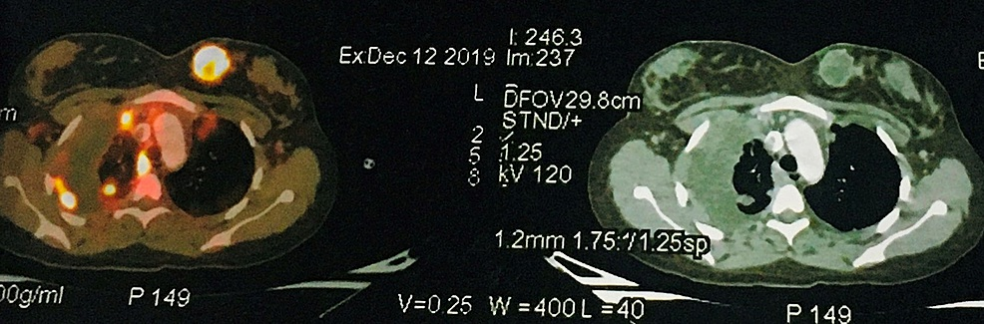
PET-CT scan showing increased fluorodeoxyglucose (FDG) uptake in the inner quadrant of the left breast of size 3.3 x 3.5 cm with SUVmax = 20.9 SUVmax: maximum standardized uptake value

**Figure 5:**
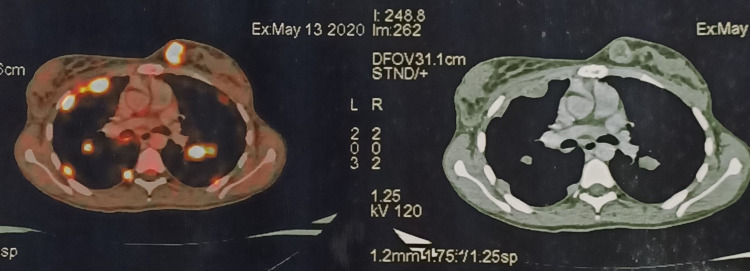
PET-CT scan showing residual disease

A 20-year-old female with unremarkable medical and surgical history presented to our institute with painful swelling over the right knee and breast lump. She had undergone patellectomy with repair three months prior at another hospital. The histopathological and immunohistochemistry (IHC) study report was suggestive of osteogenic sarcoma as it was positive for smooth muscle actin (SMA), S-100, CD68, and negative for caldesmon and desmin and CD34 (Figures 1-2). On subsequent follow up, clinical examination of the left breast showed a palpable lump of size 3 x 3 cm in the inner quadrant of the left breast (Figure 3). A whole-body PET-CT scan showed an enhancing hypermetabolic mass lesion of size 5.4 x 6.4 cm with maximum standardized uptake value (SUVmax) = 29.2 in the periarticular and intraarticular region of the right knee. Another hypermetabolic soft tissue mass of size 3.3 x 3.5 cm with SUVmax= 20.9 was found in the inner quadrant of the left breast (Figure 4). Multiple metastatic lymph nodes were found in bilateral axilla, mediastinum, retroperitoneum, inguinal region along with hepatic and pulmonary metastasis. Ultrasonography-guided core needle biopsy from breast lump showed matrix producing poorly differentiated malignant neoplasm with epitheloid and spindle cell features with necrosis. IHC showed cytokeratin, vimentin, FLI-1- moderate to strong positive, S-100 and SMA - focally positive, GATA binding protein 3 (GATA-3), CD34, CD31 negative, and mindbomb homolog 1 (MIB-1) index-70% which further suggested osteosarcoma metastasis to breast. The patient was subsequently advised six cycles of injection gemcitabine and docetaxel chemotherapy. Response assessment following chemotherapy showed residual disease with a partial response on PET-CT scan (Figure 5). Currently, the patient is on second-line chemotherapy injection doxorubicin and cisplatin.

## Discussion

Osteosarcoma is the most common skeletal malignancy [1]. It most commonly involves the appendicular skeleton-like metaphysis of the distal femur and proximal tibia and humerus. Metastasis occurs in 30% of patients with osteosarcoma [6-7] and most commonly metastasize to the lung followed by the skeleton [8-9]. The clinically detectable lymph node metastasis is uncommon - only 2.3% to 10% [10-11] and also metastasis to the breast is extremely rare. The most common sites of lymph node metastasis are mediastinal, inguinal, and retrocrural but axillary lymph node metastasis are also very rare, as seen in our case. The prognosis of metastatic patients remains poor and also extra pulmonary metastasis at initial presentation is an independent predictive factor of poor survival [12].

The incidence of breast metastasis from extra mammary malignancy is very low [13-14]. Among these commonly reported sites of origin in the literature are the lung, stomach, ovary, and skin [15-16]. Mostly breast metastasis is detected incidentally by radiological imaging. But in our case, there was a clinically palpable breast lump which was hard, nontender, and mobile in the lower and inner quadrant. Additionally, IHC study also helps in differentiating metastatic osteosarcoma to breast from primary breast lesion. In our case, the breast lesion showed IHC positive to s100, vimentin, special AT-rich sequence-binding protein 2 (SATB2), and SMA positive which are commonly positive in osteosarcoma. Again IHC showed negative to GATA3 which exclude the diagnosis of primary breast malignancy.

There is no specific guideline for the management of this extremely rare case, so osteosarcoma with breast metastasis is treated the same as metastasis to other sites. The general treatment strategy for osteosarcoma is complete surgical excision and systemic chemotherapy, both neoadjuvant and adjuvant. Palliative chemotherapy should be considered in case of extensive metastasis. So the treatment protocol in our case is palliative chemotherapy due to extensive metastasis.

## Conclusions

Incidence of breast metastasis in osteosarcoma of the knee is very rare. Histopathology features and IHC study help to differentiate metastatic osteosarcoma to the breast from primary breast malignancy. Due to the rarity of cases, no specific diagnostic and management strategy has been found for these types of cases. So by reporting this extremely rare case of osteosarcoma of the right knee with breast and axillary lymph node metastasis, we recommend vigilance in metastatic work up and treatment of metastatic osteosarcoma.
